# A Systematic Review of Ethanol and Fomepizole Use in Toxic Alcohol Ingestions

**DOI:** 10.1155/2013/638057

**Published:** 2013-01-31

**Authors:** Lorri Beatty, Robert Green, Kirk Magee, Peter Zed

**Affiliations:** ^1^Department of Emergency Medicine, Dalhousie University, Room 377, Bethune Building, 1276 South Park Street, Halifax, NS, Canada B3H 2Y9; ^2^Division of Critical Care Medicine, Department of Anesthesia, Dalhousie University, Room 377, Bethune Building, 1276 South Park Street, Halifax, NS, Canada B3H 2Y9; ^3^Faculty of Pharmaceutical Sciences, University of British Columbia, Vancouver, BC, Canada V6T 1Z3

## Abstract

*Objectives*. The optimal antidote for the treatment of ethylene glycol or methanol intoxication is not known. The objective of this systematic review is to describe all available data on the use of ethanol and fomepizole for methanol and ethylene glycol intoxication. *Data Source*. A systematic search of MEDLINE and EMBASE was conducted. *Study Selection*. Published studies involving the use of ethanol or fomepizole, or both, in adults who presented within 72 hours of toxic alcohol ingestion were included. Our search yielded a total of 145 studies for our analysis. There were no randomized controlled trials, and no head-to-head trials. *Data Extraction*. Variables were evaluated for all publications by one independent author using a standardized data collection form. *Data Synthesis*. 897 patients with toxic alcohol ingestion were identified. 720 (80.3%) were treated with ethanol (505 Me, 215 EG), 146 (16.3%) with fomepizole (81 Me, 65 EG), and 33 (3.7%) with both antidotes (18 Me, 15 EG). Mortality in patients treated with ethanol was 21.8% for Me and 18.1% for EG. In those administered fomepizole, mortality was 17.1% for Me and 4.1% for EG. Adverse events were uncommon. *Conclusion*. The data supporting the use of one antidote is inconclusive. Further investigation is warranted.

## 1. Introduction

Toxic alcohol poisonings with methanol or ethylene glycol have the potential to cause significant morbidity and mortality. In 2009, poison centers in the United States (US) received 8139 reports of toxic alcohol ingestion of which 29 died, and 259 had a major outcome (defined as life threatening, or resulting in significant residual disability) [1]. Both methanol (Me) and ethylene glycol (EG) are metabolized by the liver enzyme alcohol dehydrogenase (ADH) to toxic metabolites, which cause a profound metabolic acidosis, along with other serious toxic effects.

The mainstay of treatment for both Me and EG ingestion is the administration of an antidote which blocks the function of ADH, thereby preventing the formation of toxic metabolites. Patients may also require correction of their metabolic acidosis and electrolyte abnormalities, and hemodialysis. Currently there are two antidotes used to block ADH metabolism: ethanol, a competitive ADH substrate, and fomepizole, an ADH inhibitor.

Fomepizole is the most commonly administered antidote in the management of toxic alcohol ingestions in the US. It was first approved for use in the US for the treatment of EG toxicity in 1997, and for the treatment of methanol toxicity in 2000. In 2009, among cases reported to US poison centres, fomepizole was used in 1743 cases of toxic alcohol ingestion, compared to only 96 cases in which ethanol was used [1]. In comparison, poison control data from 2000 indicate that fomepizole was used in only 167 poisonings, while ethanol use was reported 305 times [2]. Despite this change in the management of patients with toxic alcohol ingestion, there has been no direct comparison of ethanol and fomepizole, in terms of efficacy, safety, or cost-effectiveness to provide evidence that one antidote is superior to the other. The objective of this paper is to perform a systematic review of the literature to describe the efficacy and safety of ethanol and fomepizole as an antidote in the treatment of methanol and ethylene glycol intoxication. Specifically, we sought to determine differences in clinically important outcomes and adverse events (AE) in adult patients treated with fomepizole or ethanol after ingestion of a toxic alcohol, in the published medical literature.

## 2. Methods

### 2.1. Data Source

A systematic search of MEDLINE (1966 to August 2010) and EMBASE (1974 to August 2010) was conducted for full-text reports. All articles describing the treatment of an acute methanol or ethylene glycol ingestion with ethanol, fomepizole, or both were evaluated. The search terms included “methanol” or “methyl alcohol” or “wood alcohol” or “ethylene glycol” or “2-hydroxyethanol” or “monoethylene glycol” or “antifreeze” and “fomepizole” or “4-methylpyrazole” or “ethanol” or “ethyl alcohol” (see Appendix B).

### 2.2. Study Selection

Citations identified following the literature search were evaluated for relevance on the basis of title and abstract. The full texts of relevant articles were evaluated independently by two authors using predetermined inclusion criteria. Agreement was measured using simple agreement statistics. Where question remained regarding eligibility for inclusion, the author of the publication in question was contacted for more details.

Predetermined inclusion criteria were as follows: clinical trials; case series and case reports were included if they described an acute ingestion of methanol or ethylene glycol in adult patients (age ≥18 years); administration of ethanol or fomepizole (or both); and at least one clinically relevant outcome, which included admission to hospital, admission to intensive care unit (ICU), intubation, dialysis, visual impairment, renal impairment, complications related to treatment, death, or other adverse outcomes. Reports describing animal studies, transcutaneous, or inhalational exposure to toxic alcohols and those available only as abstracts were excluded. After study identification, only papers published in English of French were included.

### 2.3. Data Extraction

Data elements were evaluated for all publications independently by a single author using a standardized data collection form. Ten percent of included cases were randomly selected and the data was extracted by a second investigator and compared with the initial data collection form to evaluate the quality of the data extraction. The authors involved in study selection and data extraction were not blinded to the purpose of the study. Primary data elements included the following: year, country of publication, patient age and gender, amount of toxic alcohol ingested, serum toxic alcohol level, serum ethanol level, presence of coingestion, serum pH, time to treatment, antidote dose and protocol, and duration of dialysis, where applicable. Clinically relevant outcomes included mortality, hospital admission, admission to an ICU, hospital and ICU length of stay (LOS), and renal or visual impairment or need for hemodialysis. Adverse events included hypoglycemia, seizure, intubation related AE's, dialysis related AE's, pneumonia, dysrhythmia, and pancreatitis. 

 Only data explicitly stated in the paper of included studies was extracted. Nonexplicit data was treated as missing, and analysed as such. No data was imputed.

### 2.4. Definitions

For the purposes of this study, serum toxic alcohol level, serum ethanol level, and serum pH represent the first level reported after patient presentation to hospital, and before any therapeutic ethanol was administered. If a value was reported as mg/dL units, the value was converted to mmol/L using the following formulae: ethylene glycol-mmol/L = 0.1611 × mg/dL; methanol-mmol/L = 0.312 × mg/dL; and ethanol-mmol/L = 0.217 × mg/dL as per the AMA Author's Guide [3]. Patients were considered to have an ethanol co-ingestion if the patient admitted to ingesting ethanol prior to hospital presentation, or if the initial pretreatment serum ethanol level was positive. Other coingestions included other medications and recreational substances ingested by the patient, excluding tobacco and ethanol, either reported by patient, bystanders, or prehospital care providers. Time to diagnosis represents time from reported patient ingestion to time to initial treatment.

Route of administration of ethanol was recorded only if specifically reported in the study. For fomepizole dosing, a “standard” regimen was considered to be the administration of intravenous fomepizole: 15 mg/kg then 10 mg/kg every 12 hours for 4 doses; then 15 mg/kg every 12 hours until ME or EG level was normal, acidosis had cleared, and patient was symptom-free; and 10 mg/kg every 4 hours while on dialysis [4–6]. Anything other than the above regimen was considered a “nonstandard” regimen. Data on intubation was collected only if explicitly stated in the article that patient was or was not intubated.

Patient death was recorded if the patient died during initial hospital admission. Patients were considered to be admitted to hospital if the article reported admission to hospital or if the patient received dialysis or required intubation. Length of admission was recorded as the number of days from patient presentation to discharge home, death, or transfer to rehab centre or psychiatric care. Patients were considered to be admitted to ICU if the article reported admission to ICU or if the patient required intubation. Length of ICU admission was recorded as number of days from ICU admission to death or transfer to home, a medical floor, or another institution. Patients were considered to have undergone dialysis if they received either hemo- or peritoneal dialysis for initial treatment of toxic alcohol ingestion or for renal insufficiency. Renal impairment was defined as an elevation of creatinine above baseline at the time of hospital discharge. Visual impairment was defined as any visual deficit at the time of discharge (provided it was not a preexisting condition). Only adverse events documented by authors are reported.

### 2.5. Data Analysis

In most cases, individual case data was available and collected as such. When only aggregate data was presented in the article, it was collected and combined with the individual data for analysis whenever possible. Descriptive statistics were calculated for all of the above-mentioned variables where individual case data could be collected, subgrouped by toxin ingested and antidote given. Categorical variables were described in terms of percentage, and continuous variables were described as mean ± standard deviation. Median values for continuous variables were also calculated. All calculations were performed using Microsoft Excel 2007 (Microsoft Corporation, Redmond, WA). Due to significant heterogeneity in study designs and populations, a meta-analysis of the data was not possible.

## 3. Results

**Figure 1:**
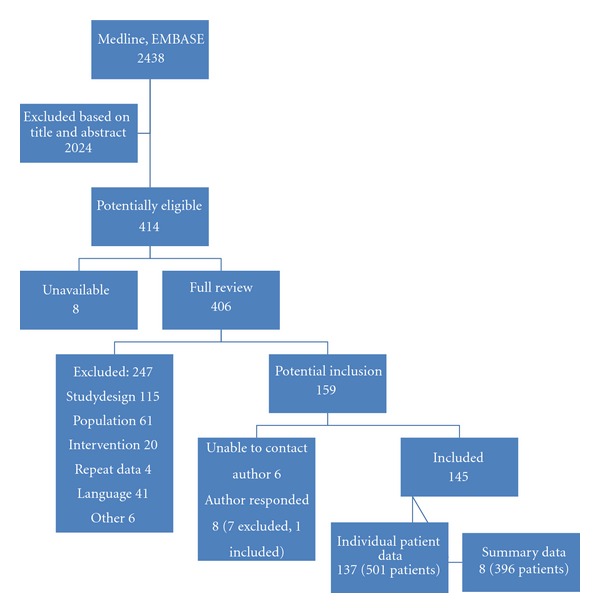


A total of 2438 articles were identified by the search, and 145 met inclusion criteria, including 137 case reports or case series presenting individual data [6–143], and 8 case series presenting aggregate data only (Figure 1) [5, 144–150]. There were no randomized controlled trials. A total 897 patients with toxic alcohol ingestion were identified, with 501 cases with individual patient data, and 396 cases with aggregate data. Overall 602 patients with methanol ingestion (67.1%), 293 with ethylene glycol ingestion (32.7%), and 2 patients who ingested both Me and EG (0.2%) were included, resulting in a total of 899 exposures. Of these patients, 720 (80.3%) were treated with ethanol (505 Me, 215 EG), 146 (16.3%) were treated with fomepizole (81 Me, 65 EG), and 33 (3.7%) were treated with both antidotes (18 Me, 15 EG).

Patient demographics are presented in Table 1 (individual case data) and Table 2 (aggregate data). The mean (standard deviation) age among cases presented in the literature was 41.3 years (±14.1 years) and the majority were male (79.6%). Serum toxic alcohol level in subjects varied between treatment groups (EG 20.3–46.2 mmol/L, Me 43.8–63.0 mmol/L). The time to diagnosis ranged from 0.5 to 72 hours, but was generally longer in Me patients than EG patients. Initial serum pH was reduced in all groups (7.13–7.26). A total of 17 EG patients and 38 Me patients reported co-ingestion of ethanol, and 4 EG patients and 3 Me patients reported other co-ingestions.

A standard fomepizole dosing protocol was used in 70% and 39% of Me and EG cases, respectively (Table 3). In cases treated with ethanol, 2.7% of EG and 11.1% of Me were treated with oral ethanol, 87.7% of EG and 30.2% of Me patients received IV ethanol, and 0.7% of EG and 0.8% of Me patients received both IV and oral ethanol.

Overall, the requirement for intubation was underreported, with intubation status described in 223 of 897 (24.9%) patients (Table 3). Intubation rates, when reported, appeared high in all three antidote groups: ethanol (80/153, 52.3% Me; 26/33, 78.8% EG), fomepizole (7/12, 58.3% Me; 8/15, 53.3% EG), and both (3/7, 42.9% Me; 1/3, 33.3% EG). When toxin was considered, intubation rates were relatively low following treatment in patients who ingested EG (4/25, 16% ethanol; 1/8, 12.5% fomepizole; 0/1, 0% both), and in those who ingested methanol (3/19, 15.8% ethanol; 0/7, 0% fomepizole; 2/3, 66.7% both). 

Mortality in patients treated with ethanol was 21.8% for Me and 18.1% for EG, and in those administered fomepizole was 17.1% for Me and 4.1% for EG. The mortality in patients treated with both antidotes was 5.5% for Me and 7.1% for EG (Table 4). Admission to hospital was reported in all cases, yet admission to ICU was infrequently reported (11.5%). Amongst the papers that reported ICU admission, ICU admission was high (>80%). Mean hospital length of stay ranged from 4 to 12.6 days. Mean ICU length of stay, though infrequently reported, ranged from 4 to 11.5 days. Hemodialysis was instituted in >65% of patients in all groups. Among patients who ingested EG, renal impairment was reported more frequently in patients treated with ethanol (59/149, 39.6%) than those treated with fomepizole (5/36, 13.9%). Among patients who ingested methanol, rate of visual impairment was 18.2% in the ethanol group, 18.1% in the fomepizole group, and 23.1% in the patients who receive both ethanol and fomepizole. 

Very few adverse events were reported in patients treated with either fomepizole or ethanol (Table 4). Hypoglycemia was described only twice (2/897, 0.2%) among the case reports, only in patients receiving both antidotes. The most commonly reported adverse events included pneumonia (8/897, 0.9%, all in patients given ethanol), pancreatitis (7/897, 0.8%, one case series of seven patients who all received ethanol), and seizure in four patients treated with ethanol and three treated with fomepizole (4/720, 0.6% ethanol; 3/146, 2% fomepizole). The overall number of cases reporting need for intubation was very small.

Agreement between investigators on data extraction was 96%. 

## 4. Limitations

We acknowledge several limitations of our systematic review. As with all systematic reviews, it is possible that some reported cases in the literature were missed. This risk was limited by using a systematic search strategy in collaboration with a professional health services librarian with extensive experience in systematic reviews. We included a number of terms for each alcohol in the search strategy, did not place any limits on the search, and searched multiple databases. In addition, all articles were reviewed by more than one reviewer to determine inclusion. Because of resource limitations, we only collected data from studies that were published in English or French, opening our study to language bias. However, exclusion of papers in other languages was not done until after our search, allowing the research team to review the number of cases published in other languages. A total of 41 papers were excluded based on language, containing a total of 51 patients. The majority of these papers were from western European countries. As this would account for approximately 5% of the total number of patients analyzed, we feel that the exclusion of these publications is unlikely to significantly impact our findings.

This review is further limited by the lack of any randomized controlled trials or even cohort trials in the published literature, which precluded any direct, head-to-head comparisons. The current available literature on methanol and ethylene glycol ingestions exists largely in the form of case reports and case series, in which patients are not uniform, reporting of ingestions may not be accurate, and timing and quality of interventions are not documented in real time. The fact that these papers may be further affected by recall and publication bias places a significant limitation on the conclusions we are able to draw from the data.

Furthermore, the quality of data available in these published studies is generally poor, as important data points were lacking in many of the studies. All available data was collected for analysis, but due to incomplete reporting, it is possible that our results may not reflect the true values. This issue was further complicated by the fact that more than one-third of our data was available in aggregate form only, which limits our ability to combine and summarize data. Additionally, we have noted substantial variation in baseline characteristics of the groups, including age, time to diagnosis, and serum toxic alcohol level, making any meaningful comparison of the outcome data problematic.

Potential reporting and publication biases may also limit the validity of this review. Cases of toxic alcohol ingestion without adverse outcomes may be less likely to be reported or published. This is reflected in the fact that mortality in our study ranged from 4 to 21% amongst groups, while the mortality rate in the 2009 US poison center data is only 0.4% [1]. Even after considering that poison centre data may underestimate the true mortality rate, given that many reported cases may have had a very small, if any, ingestion of the suspected alcohol, the mortality rate found in our study appears high. Thus, the results of this review may be skewed toward higher complication rates, more severe acidosis, and higher mortality than may be present in the general clinical patient population.

Additionally, ethanol has been used as an antidote for toxic alcohol ingestion for at least 50 years, while fomepizole did not appear in the literature until 1986. As a result, cases describing ethanol use tend to be temporally remote, while more recent publications focus on fomepizole. Advances in supportive care in emergency medicine, intensive care medicine, and nephrology may also have a significant influence on patient outcomes, in addition to the antidote administered. Despite the limitation of publication type and data quality, the results of this study compile data on nearly 900 cases, providing the best estimates possible for the data points collected.

## 5. Conclusion

Methanol and ethylene glycol are relatively common and potentially fatal toxic alcohol ingestions. Both alcohols are metabolized in the liver by the enzyme alcohol dehydrogenase (ADH) to produce toxic metabolites which are responsible for an anion-gap metabolic acidosis, and other adverse effects. Methanol is commonly found in solvents, deicers, glass cleaners, as well as homemade alcohols (“moonshine”). It is metabolized to formic acid which can cause neurologic injury, optic nerve toxicity, and retinal injury resulting in vision impairment [151–153]. Ethylene glycol is found in antifreeze, brake fluids, and coolants and has a sweet taste which makes it attractive to animals and small children. Toxic metabolites of EG include glycolate and oxalic acid, which can cause renal failure and neurologic impairment. Unfortunately, both alcohols can be fatal even in small quantities [151]. 

The paramount management strategy in the treatment of Me and EG ingestions is to prevent toxic metabolite formation by ADH blockade. Historically, ethanol has been used as an antidote and is still standard therapy in some centres, due to its low cost and physician familiarity. However, for ethanol to be an effective antidote, most experts feel the serum level must be carefully titrated and maintained between 22 and 33 umol/L [154]. This requires frequent adjustments of the infusion, may place demands on valuable nursing time, and may carry a risk of depressed level of consciousness, agitation, hypoglycemia, pancreatitis, and further increasing serum osmolality [155]. Additionally, some hospital pharmacies do not stock the appropriate concentration of ethanol for IV administration, making timely acquisition of the antidote challenging. Fomepizole is administered as a weight-based fixed dose at regular intervals, without the need for monitoring of serum levels, and the literature to date has not reported a substantial incidence of adverse events [5, 6].

In recent years, there has been a move towards the exclusive use of fomepizole in the treatment of toxic alcohol intoxication. Some authors feel that fomepizole pharmacokinetics are more predictable than ethanol, has a safer side-effect profile, shortens ICU and hospital stays, and decreases need for hemodialysis, at least in EG patients [156–159]. As is common in toxicology, there are few high-quality clinical studies to support or refute these arguments. The evidence supporting a move toward favouring fomepizole over ethanol consists of a few small, noncomparative trials, [5, 6, 93, 159] involving a total of 82 patients, only 30 of which were prospectively studied. Although there are no clinical head-to-head studies in the literature, guideline authors consider ease of administration, pharmacokinetic predictability, product availability, side-effect profile, and laboratory and animal studies, when creating recommendations for toxic alcohol management, and many current guidelines promote the use of fomepizole over ethanol in adult and pediatric patients [160, 161]. 

This review is the most extensive in the literature to date comparing the use of ethanol and fomepizole in the management of toxic alcohol ingestion. It includes all published cases on patients given an antidote for an EG or Me ingestion, based on an exhaustive search of multiple databases. As a head-to-head comparison of the antidotes does not currently exist, it was hoped that this systematic review of the published literature would provide valuable information to aid in the treatment of this patient population.

Unfortunately, the quality of published data available for our systematic review limits the conclusions which can be drawn. A lack of consistency in reporting, including the timing of lab values, the time to investigations and treatment, and the description of patient outcomes, makes it problematic to make comparisons between groups of patients. Additionally, examination of the aggregate data shows that many of the baseline characteristics (time to diagnosis, toxic alcohol serum level, ethanol level) differ significantly between groups, thus statistical comparisons of different treatment groups cannot be done.

Although the quality of studies and data limit a direct comparison of ethanol and fomepizole, some meaningful observations can still be made. First, considering the relatively high rate of toxic alcohol exposures (8139 reported in the US in 2009), there is a paucity of cases published in the literature, and publications describing the use of fomepizole are even less common (146/897, 16.3% of published cases). Second, there is a temporal pattern in the reporting of antidote use, with the majority of reports before the mid-1990s describing ethanol use, while the more recent literature focuses mainly on fomepizole. This temporal pattern may be important, as advances in the care of these patients independent of ADH blockade strategy may significantly impact patient outcomes. Fomepizole use is much more commonly reported in recent years, when advances in general supportive emergency department care, critical care, and hemodialysis may have contributed significantly to improved patient outcome.

Some authors have suggested that the use of fomepizole may decrease the need for hemodialysis. Borron et al. published a letter describing the use of fomepizole without dialysis in 8 patients, with good outcomes [159]. Several other authors have presented case reports describing the use of fomepizole without dialysis [133, 162, 163]. The Methylpyrazole for Toxic Alcohols Study Group used EG clearance rates to suggest that serum EG level alone should no longer be used as an indication for hemodialysis [164]. They concluded that because fomepizole completely blocks formation of toxic metabolites, and because fomepizole side effects are few, patients with normal renal function should be allowed to clear the toxic alcohol and minimize the need for hemodialysis for toxin removal. Similarly, in the pediatric population Brent suggested that fomepizole may obviate the need for hemodialysis [157], based on a total of six published cases in the literature.

Despite hemodialysis being commonly considered as part of the toxic alcohol management, there is a similar lack of quality data to support the need for dialysis in the setting of poisoned patients with no acidosis and normal renal function. Some experts advocate the use of repeated doses of fomepizole in stable patients to obviate the use of hemodialysis, while others caution against the exclusion of hemodialysis in these cases, as the adverse effects of a prolonged clearance half-life of methanol or ethylene glycol in the absence of hemodialysis are not yet known. Several authors have called for further research in this area as some controversy exists on the utility of this approach. Based on our systematic review of the literature, we have found insufficient data to support or refute the use of hemodialysis in ethylene glycol and methanol ingestion. The initial pH for all groups in our review was reduced, suggesting that most patients had already begun metabolizing the toxic alcohols, and formed toxic metabolites, thus necessitating hemodialysis. This may explain the similar rates of hemodialysis among all patient groups. We feel that further research is required in order to determine whether hemodialysis can be safely omitted in the stable toxic alcohol ingestion with a normal pH and anion gap. 

Our study did find an apparent difference in uncontrolled absolute mortality between treatment groups for patients with Me and EG ingestions. In the Me group, mortality was 21.8% in those treated with ethanol, 17.1% in those treated with fomepizole, and 5.5% in those who received both. Among reported cases of EG ingestion, mortality was 18.1% in those who received ethanol, 4.1% in those given fomepizole, and 7.1% in those treated with both. The difference in baseline characteristics and quality of reported data make it unwise to conclude that the mortality difference is due to specific antidote utilization. As discussed above, a temporal publication bias, or other unmeasured confounder, may have contributed to these apparent differences in mortality. Nevertheless, the differences in mortality between groups is interesting, especially among the EG patients, and warrants further examination of the data and further research in the area to determine whether a true significant difference may exist.

Interestingly, adverse events related to the use of ethanol and fomepizole were uncommonly reported in the studies we reviewed. The prevalence of adverse events in patients with toxic alcohol ingestions has been recently reported by Lepik et al. in a 2009 retrospective chart review [165]. This study evaluated 172 cases of toxic alcohol ingestion from 10 centres in British Columbia, Canada. One hundred and thirty were treated with ethanol and 44 treated with fomepizole. They found the rate of adverse events (57% (74/130) versus 12% (5/42)) and severe adverse events (20% (26/230) versus 5% (2/42)) were more common among those treated with ethanol than those given fomepizole. CNS depression was the most frequent adverse event among ethanol-treated patients and was rare among those treated with fomepizole. Hypoglycemia was observed in 4% of ethanol-treated patients, and in none of the fomepizole patients. The high number of adverse events described in this study supports the likelihood that underreporting of events was common in the literature we reviewed. These data are not included in the data summarized in the table, because the results for patients taking EG and ME were presented in aggregate in the paper.

A review by Hantson et al. in 1997 outlines several drawbacks to the use of ethanol as an antidote [47], including the need for a central line, the challenge in maintaining an adequate serum level, hypoglycaemia, CNS depression, and behavior issues. We did not find hypoglycaemia to be a common side effect (2/897, 0.2%) reported in the adult literature, although it is possible that this has been underreported. We did find a higher rate of posttreatment intubations in the ethanol-treated patients, compared to those treated with fomepizole (7/44, 15.9% ethanol; 1/15, 6.7% fomepizole). Although the reported numbers were small, these results may suggest a more a depressed level of consciousness among ethanol-treated patients. The underreporting of adverse events in the data we analysed prevents us from drawing any conclusions regarding the safety of either antidote. 

Overall, we have identified 897 patients reported in the literature who have been treated with either ethanol or fomepizole, all in the form of case series and case reports. In general, the quality of the data is poor, with significant heterogeneity and a lack of consistency in reporting. We note little differences in important patient outcomes in the available data, such as mortality, ICU admission, and need for hemodialysis. Interestingly, very few adverse events have been reported in association with either medication.

One important consideration in the use of fomepizole and ethanol is the relative cost benefit of these antidotes. Acquisition costs are different between fomepizole and ethanol, yet the total health care costs need to be considered.

Based on our systematic assessment of the literature, we urge further research into the relative benefits of ethanol and fomepizole in the management of toxic alcohol ingestions. Until further evidence on the use of ethanol and fomepizole is available, either antidote may be considered for ADH blockade. Clinicians should consider other factors, including cost, efficiency, resource utilization, patient demographic, availability of antidote, and familiarity with the drug when making decisions on the management of toxic alcohol ingestion. 

## Figures and Tables

**Table 1:** Patient demographics; individual cases.

Variable	Ethylene glycol			Methanol		
	Ethanol	Fomepizole	Both	Ethanol	Fomepizole	Both
	**(n = 166)**	**(n = 31)**	**(n = 15)**	**(n = 201)**	**(n = 72)**	**(n = 18)**
Age (mean ± SD)	(*n* = 162)	(*n* = 21)	(*n* = 15)	(*n* = 189)	(*n* = 72)	(*n* = 18)
	42.2 ± 14.3	42.5 ± 14.7	40.6 ± 15.4	39.0 ± 13.7	44.9 ± 12.8	43.7 ± 17.2
	Median = 42	Median = 45	Median = 42	Median = 36	Median = 45	Median = 42

Gender (% male)	(*n* = 144)	(*n* = 21)	(*n* = 15)	(*n* = 199)	(*n* = 72)	(*n* = 18)
	84%	67%	80%	73%	68%	56%

Region	(*n* = 166)	(*n* = 31)	(*n *= 15)	(*n* = 201)	(*n* = 72)	(*n* = 18)
North America	19%	32%	33%	52%	29%	39%
Europe	81%	68%	67%	37%	71%	61%
Other	0%	0%	0%	11%	0%	0%

Time to diagnosis (h) (range)	(*n* = 124)	(*n* = 19)	(*n* = 7)	(*n* = 145)	(*n* = 15)	(*n* = 7)
	0.5–24	0.25–22	0.5–36	1–72	0.5–30	2–41
	Median = 2	Median = 6	Median = 11	Median = 6.3	Median = 9.5	Median = 6.5

Toxic alcohol serum level	(*n* = 113)	(*n* = 30)	(*n* = 15)	(*n* = 186)	(*n* = 72)	(*n* = 18)
(mmol/L)	24.7 ± 43.9	46.2 ± 58.9	20.3 ± 16.8	63.0 ± 61.4	43.8 ± 48.3	58.3 ± 68.2
(mean ± SD)	Median = 8.0	Median = 23	Median = 15.7	Median = 43.8	Median = 23.5	Median = 27.3

Ethanol serum level	(*n* = 19)	(*n* = 9)	(*n* = 1)	(*n* = 40)	(*n* = 57)	(*n* = 7)
(mmol/L)	20.4 ± 31.1	8.2 ± 13.6	0	6.3 ± 12.3	7.3 ± 18.6	4.4 ± 10.5
(mean ± SD)	Median = 0.03	Median = 0	Median = 0	Median = 0	Median = 0	Median = 0

Initial serum pH	(*n* = 126)	(*n* = 27)	(*n* = 12)	(*n *= 190)	(*n* = 69)	(*n* = 16)
(mean ± SD)	7.21 ± 0.22	7.16 ± 0.21	7.18 ± 0.16	7.13 ± 0.25	7.16 ± 0.27	7.26 ± 0.14
	Median = 7.29	Median = 7.17	Median = 7.18	Median = 7.19	Median = 7.23	Median = 7.27

Coingestion	(*n* = 30)	(*n* = 5)	(*n* = 1)	(*n* = 30)	(*n* = 28)	(*n* = 6)
Ethanol (*n*)	13	3	1	20	17	1
Other (*n*)	3	1	0	1	2	0

ME: methanol, EG: ethylene glycol, SD: standard deviation.

Data points were not available for every case. Calculations of mean, median, and SD were done using available data only. **n** in bold at the top of each column represents total number of patients in the group. *n* in each cell represents number of patients for whom data was present.

**Table 2:** Patient demographics; aggregate data.

Data	Variable					
	Age (years) (mean ± SD)	Gender (% male)	Time to diagnosis (hours) (mean ± SD)	Toxic alcohol level (mmol/L) (mean ± SD)	Ethanol Level (mmol/L) (mean ± SD)	pH (mean ± SD)
Ethylene Glycol						
Fomepizole						
Cases (*n =* 31)	42.5 ± 14.7	67%	8.8 ± 7.2	46.2 ± 58.9	8.2 ± 13.6	7.16 ± 0.21
Brent 1999 (*n =* 19)	41 ± 13	89%	6.6–20.8	3.87–71.85	0–39.3	6.93–7.47
Megarbane 2008 (*n =* 15)	31 (27–51)	83%		PO: 1.0 ± 11.3 IV: 6.8 ± 53.5		
Ethanol						
Cases (*n =* 166)	42.2 ± 14.3	84%	5.3 ± 8.4	24.7 ± 43.9	20.3 ± 31.1	7.21 ± 0.22
Karlson-Stiber 1992 (*n =* 32)	20–69					6.7–7.4
Hylander 1996 (*n =* 17)	38 ± 15	94%		Alive: 33 ± 38.6 Dead: 8.7 ± 6.1		Alive: 7.2 ± 0.1 Dead: 6.9 ± 0.2
Both						
Cases (*n =* 15)	40.6 ± 15.4	80%	13.3 ± 12.6	20.3 ± 16.8	0	7.18 ± 0.16

Methanol						
Fomepizole						
Cases (*n =* 72)	44.9 ± 12.8	68%	13.0 ± 10.7	43.8 ± 48.3	7.3 ± 18.6	7.16 ± 0.27
Megarbane 2008 (*n =* 9)	46 (38–55)	83%		PO: 3.12 IV: 7.49 ± 32.45		
Ethanol						
Cases (*n =* 201)	39.0 ± 13.7	73%	15.2 ± 17.2	63.0 ± 61.4	6.3 ± 12.3	7.13 ± 0.25
Krishnamurthi 1968 (*n =* 89)		87%				
Paasma 2007 (*n =* 111)				Alive: 1.3–175 Dead: 28.4–194.7		Alive: 7.14 Dead: 6.78
Moghadami 2008 (*n =* 62)	31 ± 12.7	100%		8.49 ± 9.69	5.10 ± 6.10	
Taheri 2010 (*n =* 42)	38 (15–81)	93%	48.6 (24–72)	9.39 (6.86–20.9)		6.9 (6.3–7.2)
Cases (*n =* 18)	43.7 ± 17.2	56%	11.5 ± 13.9	58.3 ± 68.2	4.4 ± 10.5	7.26 ± 0.14
Both						
Cases (*n =* 18)	43.7 ± 17.2	56%	11.5 ± 13.9	58.3 ± 68.2	4.4 ± 10.5	7.26 ± 0.14

**Table 3:** Treatment and intubation data by EG and Me.

Variable	Ethylene glycol			Methanol		
	Ethanol	Fomepizole	Both	Ethanol	Fomepizole	Both
	**(n = 215)**	**(n = 65)**	**(n = 15)**	**(n = 505)**	**(n = 81)**	**(n = 18)**
Regimen^1^	(*n* = 146)	(*n* = 59)	(*n* = 5)	(*n* = 251)	(*n* = 74)	(*n* = 13)
Fomepizole						
Standard		39%	0%		70.3%	53.8%
Other		59%	100%		29.7%	38.5%
Ethanol						
Oral only	2.7%		20%	11.1%		0%
IV only	87.7%		20%	30.2%		38.5%
Oral and IV	0.7%		20%	0.8%		0%

Intubation	(*n* = 33)	(*n* = 15)	(*n* = 3)	(*n* = 153)	(*n* = 12)	(*n* = 7)
(% of total reported)	78.8%	53.3%	33.3%	52.3%	58.3%	42.9%

Intubation after ADH blockade	(*n* = 25)	(*n* = 8)	(*n* = 1)	(*n* = 19)	(*n* = 7)	(*n* = 3)
(% of intubated patients)	16%	12.5%	0%	15.8%	0%	66.7%

ME: methanol, EG: ethylene glycol, IV: intravenous, ADH: alcohol dehydrogenase.

Results are a combination of data from individual case data and aggregate data. **n** in bold at top of each column represents total number of patients in the group. *n* in each cell represents number of patients for whom data was present.

^1^Percentages do not total 100% because some cases did not describe the route of ethanol dose.

**Table 4:** Clinical outcomes by EG and Me and treatment.

Variable	Ethylene glycol			Methanol		
	Ethanol	Fomepizole	Both	Ethanol	Fomepizole	Both
	**(n = 215)**	**(n = 65)**	**(n = 15)**	**(n = 505)**	**(n = 81)**	**(n = 18)**
Death (%)	(*n* = 149)	(*n* = 49)	(*n* = 14)	(*n* = 490)	(*n* = 70)	(*n* = 18)
	18.1%	4.1%	7.1%	21.8%	17.1%	5.5%

Admission to hospital (%)	(*n* = 113)	(*n* = 47)	(*n* = 14)	(*n* = 341)	(*n* = 70)	(*n* = 18)
	100%	100%	100%	100%	100%	100%

Hospital LOS (d) (mean ± SD)	(*n* = 41)	(*n* = 8)	(*n* = 5)	(*n* = 39)	(*n* = 9)	(*n* = 5)
	12.6 ± 14.7	11.1 ± 9.6	4.0 ± 4.2	10.4 ± 18.3	5.3 ± 3.9	4.4 ± 2.2
	Median = 6.5	Median = 9	Median = 1	Median = 4	Median = 4	Median = 5

Admission to ICU (%) (mean ± SD)	(*n* = 27)	(*n* = 13)	(*n* = 9)	(*n* = 38)	(*n* = 10)	(*n* = 7)
	96.3%	84.6%	100%	81.2%	100%	85.7%

ICU LOS (d)	(*n* = 6)	(*n* = 3)	(*n* = 3)	(*n* = 6)	(*n* = 1)	(*n* = 1)
	7.3 ± 4.9	4.8 ± 2.8	5 ± 2.6	11.5 ± 7.8	8	4
	Median = 6.5	Median = 4	Median = 4	Median = 10.5	Median = 8	Median = 4

Dialysis (%)	(*n* = 209)	(*n* = 50)	(*n* = 15)	(*n* = 367)	(*n* = 72)	(*n* = 18)
	65.6%	76.0%	86.7%	85.0%	69.4%	83.3%

Renal impairment (%)	(*n* = 149)	(*n* = 36)	(*n* = 10)	(*n* = 241)	(*n* = 29)	(*n* = 8)
	39.6%	13.9%	60%	3.3%	3.4%	0%

Vision impairment (%)	N/A	N/A	N/A	(*n* = 347)	(*n* = 44)	(*n* = 13)
				18.2%	18.1%	23.1%

Adverse effect^1^	(*n* = 215)	(*n* = 65)	(*n* = 15)	(*n* = 505)	(*n* = 81)	(*n* = 18)
Hypoglycemia	0	1	0	0	0	1
Intubation related	0	0	0	1	0	0
Dialysis related	0	1	0	1	0	0
Pneumonia	4	0	0	4	0	0
Seizure	4	3	0	0	0	0
Arrhythmia	2	1	0	0	0	0
Pancreatitis	0	0	0	7	0	1

ME: methanol, EG: ethylene glycol, ICU: intensive care unit, LOS: length of stay.

Results are a combination of data from individual case data and aggregate data. **n** in bold at top of each column represents total number of patients in the group. *n* in each cell represents number of patients for whom data was present.

^1^Adverse events are reported simply as the number of incidents reported in the articles and were defined as follows:

(i) hypoglycemia: any mention of hypoglycemia after treatment was initiated (as defined by article author);

(ii) intubated related: one patient with right mainstem intubation resulting in left lung collapse;

(iii) dialysis related: one episode of thrombosis of dialysis catheter requiring urokinase, one episode of PD catheter displacement;

(iv) pneumonia: as reported by authors;

(v) seizure: as reported by authors;

(vi) arrhythmia: includes atrial fibrillation, ventricular fibrillation, and bradycardia (2 episodes in 1 patient);

(vii) pancreatitis: as reported by authors.

## Appendices

### A. Figures and Tables

For more details, see Tables 1, 2, 3, and 4 and Figure 1.

### B. Search Strategy

#### B.1. MEDLINE

((“Methanol” [Mesh]) OR (“2-Propanol” [Mesh]) OR (“1-Propanol” [Mesh]) OR (“Ethylene Glycol” [Mesh]) OR (“Ethylene Glycols” [Mesh:noexp]) OR (methanol OR wood alcohol OR methyl alcohol OR ethylene glycol OR antifreeze OR 2-hydroxyethanol OR monoethylene glycol)) AND (("Ethanol" [Mesh] OR ethyl alcohol) OR (“fomepizole” [Substance Name] OR fomepizole)) AND ((Humans [Mesh]) AND (Clinical Trial [ptyp] OR Meta-Analysis [ptyp] OR Randomized Controlled Trial [ptyp] OR Review [ptyp] OR Case Reports [ptyp] OR Clinical Trial, Phase I [ptyp] OR Clinical Trial, Phase II [ptyp] OR Clinical Trial, Phase III [ptyp] OR Clinical Trial, Phase IV [ptyp] OR Comparative Study [ptyp] OR Controlled Clinical Trial [ptyp] OR Multicenter Study [ptyp] OR ("Epidemiologic Studies" [Mesh]))) [770].

#### B.2. EMBASE

(“methanol”/exp OR “methanol” AND [humans]/lim OR (“2 propanol”/exp OR “2 propanol” AND [humans]/lim) OR (“ethylene glycol”/exp OR “ethylene glycol” AND [humans]/lim) OR (“wood alcohol”/exp OR “wood alcohol” OR “methyl alcohol”/exp OR “methyl alcohol” OR “rubbing alcohol” OR “isopropanol”/exp OR “isopropanol” OR “propanol”/exp OR “propanol” OR “2 hydroxyethanol” OR 2hydroxyethanol OR “monoethylene glycol” OR antifreeze AND [humans]/lim) AND (“alcohol”/exp OR “alcohol” AND [humans]/lim OR (“ethanol”/exp OR “ethanol” OR “ethyl alcohol”/exp OR “ethyl alcohol” AND [humans]/lim) OR (“4 methylpyrazole”/exp OR “4 methylpyrazole” AND [humans]/lim) OR (“fomepizole”/exp OR “fomepizole” AND [humans]/lim))) AND (case AND (“control”/exp OR “control”) AND (“study”/exp OR “study”) OR cohort AND (“study”/exp OR “study”) OR cross AND sectional AND (“study”/exp OR “study”) OR case AND report OR clinical AND trial OR randomised AND (“control”/exp OR “control”) AND trial OR comparative AND (“study”/exp OR “study”) OR “meta analysis”/exp OR “meta analysis” OR “review”/exp OR “review”) [1668].
